# Apolipoprotein M

**DOI:** 10.1186/1476-511X-3-21

**Published:** 2004-10-04

**Authors:** Guanghua Luo, Xiaoying Zhang, Peter Nilsson-Ehle, Ning Xu

**Affiliations:** 1 Department of Clinical Chemistry, Institute of Laboratory Medicine, University Hospital of Lund, S-221 85 Lund, Sweden; 2 Laboratory of Molecular Medicine, The Third Affiliated Hospital, Su Zhou University, Chang Zhou 213003, China

**Keywords:** Apolipoprotein M, Lipoprotein metabolism, Leptin, Hepatocyte nuclear factor-1α, Diabetes, Obese

## Abstract

Apolipoprotein M (apoM) is a 26-kDa protein that is mainly associated with high-density lipoprotein (HDL) in human plasma, with a small proportion present in triglyceride-rich lipoproteins (TGRLP) and low-density lipoproteins (LDL). Human apoM gene is located in p21.31 on chromosome 6 (chromosome 17, in mouse). Human apoM cDNA (734 base pairs) encodes 188-amino acid residue-long protein. It belongs to lipocalin protein superfamily. Human tissue expression array study indicates that apoM is only expressed in liver and in kidney and small amounts are found in fetal liver and kidney. *In situ *apoM mRNA hybridization demonstrates that apoM is exclusively expressed in the hepatocytes and in the tubule epithelial cells in kidney. Expression of apoM could be regulated by platelet activating factor (PAF), transforming growth factors (TGF), insulin-like growth factor (IGF) and leptin *in vivo *and/or *in vitro*. It has been demonstrated that apoM expression is dramatically decreased in apoA-I deficient mouse. Hepatocyte nuclear factor-1α (HNF-1α) is an activator of apoM gene promoter. Deficiency of HNF-1α mouse shows lack of apoM expression. Mutations in HNF-1α (MODY3) have reduced serum apoM levels. Expression of apoM is significantly decreased in leptin deficient (*ob/ob*) mouse or leptin receptor deficient (*db/db*) mouse. ApoM concentration in plasma is positively correlated to leptin level in obese subjects. These may suggest that apoM is related to the initiation and progression of MODY3 and/or obesity.

## Cloning and characterization of human apoM

Human apolipoprotein M (apoM) was found and initially isolated from chylomicrons by Xu and Dahlbäck in 1999 [1]. When they performed SDS-PAGE for delipidated human triglyceride-rich lipoprotein (TGRLP) and sequenced protein bands ranging from 6–45 kDa, one of sequences identified as the N-terminal sequence of MFHQIWAALLYFYGI. No homologous protein was identified in public databases, but several human expressed sequence tags (EST) were found similar to these N-terminal amino acid sequence. Based on these sequences, full-length cDNA of the novel protein was obtained with 188 amino acids [1]. Rabbit antibodies were raised against five synthetic peptides based on the protein sequence. The pooled antisera were used to analyze distribution of the protein among various lipoprotein subclasses using Western blotting. Under reducing conditions, a 26-kDa band was particularly predominant in high density lipoprotein (HDL) but was also observed in low density lipoprotein (LDL) and TGRLP. A less pronounced band (approximately 23-kDa) was observed, which corresponded in size to the non-glycosylated protein [1]. As majority of the protein is associated with lipoprotein in plasma, it fulfills the criteria for classification as an apolipoprotein. And this novel protein was named apolipoprotein M (apoM) [1] as the last previously identified apolipoprotein was called apoL [2]. Gel filtration of plasma showed that apoM was associated with HDL-sized particles in wide-type and apoA-I deficient mice and with HDL- and LDL-sized particles in LDL receptor-deficient mice, whereas it was mainly found in VLDL (very low density lipoprotein)-sized particles in high-fat, high-cholesterol-fed apoE deficient mice [3]. These data suggest that apoM mainly associates with HDL in normal mice, but also with the pathologically increased lipoprotein fraction in genetically modified mice.

## Gene location and amino acid sequence of apoM

**Figure 1:**
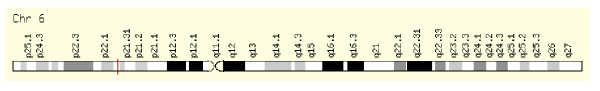
*ApoM gene location in chromosome 6*. ApoM gene is located in chromosome 6 p21.31 .

**Figure 2:**
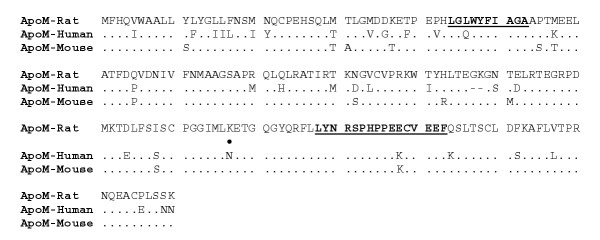
*Comparison of apoM amino acid sequence of rat, human and mouse*. Dots indicate residues that are identical to the top line (rat). One potential site for N-linked glycosylation site (Asn-Glu-Thr) is indicated by a large dot above the sequence of human apoM (○). Two sequences underlined indicate the typical lipocalin motifs.

The identified human apoM cDNA (734 base pairs) encoded 188-amino acid residue-long protein. The 5'-untranslated region was 33 nucleotides and the 3'-untranslated region 120 nucleotides, not including the poly (A) tail. Southern blot analysis of different species gave positive signals in all mammalian genomes but not in DNA from chicken and yeast [1]. Human apoM gene is located in p21.31 on chromosome 6 (Fig. 1) (chromosome 17, in mouse). The genomic sequence of this region was determined and the human apoM gene identified (GenBank accession number AF118393). In human genome, the apoM gene is surrounded by BAT4 and NG34 on one side and BAT3 on the other. Both mouse apoM gene and its human counterpart are predicted to contain 6 exons enclosed in a 1.6-kb genomic region, which is consistent with the results of Southern blotting. The calculated molecular mass of the protein was 21,256. There is one potential site for N-linked glycosylation at Asn-135 (Asn-Glu-Thr), whereas Asn-148 (Asn-Arg-Ser-Pro) is less likely to be glycosylated because Pro-151 follows Ser-150. The amino acid sequences of human and mouse apoM are 79% identical (82%, human and rat apoM) (Fig. 2), and just like human apoM the mouse sequence predicts the presence of a signal anchor, as there is no predicted signal peptidase cleavage site. The amino acid sequence of apoM contained six cysteines, which may involve in the formation of three disulfide bridges.

## Protein structure of apoM

Based on sensitive sequence searches, it is proposed that apoM is related to the lipocalin protein superfamily (Fig. 2) [1]. Subsequently, Duan et al. [4], used computer protein modeling of two lipocalins, mouse major urinary protein (MUP) and human retinol binding protein (RBP) as initial templates to build apoM protein structure, which demonstrated that apoM has the same structure of lipicalin protein superfamily. ApoM retains an uncleaved N-terminal signal peptide that most likely anchors the molecule into single layer lipids on HDL [4]. The major phospholipids in HDL is phosphatidylcholine, which has a positively charged choline group exposed to the solvent. Two electronegative regions are striking in the apoM model and are located around the N-terminus and the opening of the binding pocket. In this three-dimensional model, characterized by an eight-stranded anti-parallel β-barrel, a segment including Asn-135 could adopt a closed or open conformation. ApoM presents three disulfide bridges, which would make it a member of the lipcalin subgroup of proteins with three s-s bonds [4].

## Tissue distribution and cellular expression of apoM

Northern blot analyses of multiple tissues (including spleen, thymus, prostate, testis, ovary, small intestine, colon, leukocytes, heart, brain, placenta, lung, liver, skeletal muscle, kidney, pancreas, stomach, thyroid, spinal cord, lymph node, trachea, adrenal gland and bone marrow) showed that apoM was mainly expressed in kidney and liver [1]. Furthermore, human tissue expression array study indicated that apoM is only expressed in liver and in kidney and small amounts were found in fetal liver and in fetal kidney [5]. To elucidate whether and when apoM is expressed, Zhang et al. investigated apoM expression patterns during mouse and human embryogenesis [6]. ApoM transcripts were detectable in mouse embryos day 7.5 to day 18.5. It was expressed at low levels at day 7.5, increased significantly at day 9.7 and decreased at day 10.5, and then increased continually up to day 18.5. ApoM-positive cells appeared mainly in liver of day 12 embryos as detected by *in situ *hybridization. In day-15 embryos, apoM was expressed in both liver and kidney. During human embryogenesis, apoM was strongly expressed in livers of 3–5 month-old human embryos and continued to be strongly expressed throughout embryogenesis. In the kidney, apoM expression was highest in 5–9 month-old embryos. There was some expression of apoM in small intestine, particularly in later stages of embryogenesis. In skeletal muscle, minute apoM expression was found in 3–5 months-old embryos, and some apoM expression was found in stomach in earlier stages of embryogenesis [6]. These finding suggest that apoM has high organ specificity and strongly indicate that the physiological function of apoM must be related with liver and kidney. Both immunohistochemical staining and *in situ *apoM mRNA hybridization demonstrated that apoM is exclusively expressed in the hepatocytes and in the tubule epithelial cells in human kidney [5]. Thus, apoM may have specific function *in vivo*, which may be related to the hepatic lipid and/or lipoprotein metabolism.

## Regulation of apoM expression

*In vitro*, several biological factors have been tested to examine their influences over the transcription and secretion of apoM in hepatic cell line (HepG2 cells). Like apoB, apoM is highly hydrophobic and must co-circulate with lipoprotein particles in the blood stream. It has been demonstrated that apoB could be down-regulated by transforming growth factor-beta (TGF-β) [7,8]. Xu et al reported that TGF-β could also down-regulate apoM expression and secretion in HepG2 cells [9]. It suggests that apoM, similar to apoB, may involve in the hepatic lipoprotein metabolism *in vivo*. In another study, Xu et al demonstrated that platelet-activating factor (PAF) could up-regulate apoM expression in HepG2 cells, whereas, lexipafant, a PAF-receptor antagonist significantly suppressed the mRNA levels and the secretion of apoM in HepG2 cells in a dose-dependent manner. Neither tumor necrosis factor-α (TNF-α) nor interleukin-1α (IL-1α) influences apoM expression or secretion in HepG2 cell cultures [10]. It indicates that apoM may relate to the host defense response because apoM gene is located in histocompatibility complex III (HMC-III) region on chromosome 6. Many genes in this region are related to the immune response, and the apoM gene is very close to the TNF-α gene and lymphotoxin genes. Thus, apoM may also be related to the immune response system, or regulated by cytokines or other inflammatory factors.

Administration of adrenocorticotropic hormone (ACTH) has beneficial effects on plasma lipoproteins [11-15]. A consistent decrease of plasma total cholesterol and LDL cholesterol by 20–40% is seen during ACTH treatment [14-17]. It has been demonstrated that pronounced hypolipidimic effects of ACTH might be related to the inhibition of apoB synthesis in hepatic cells [18]. However ACTH didn't influence apoM expression and secretion *in vivo *and *in vitro *[18,19], indicates that apoM may have somewhat difference from apoB on lipid and/or lipoprotein metabolism *in vivo*. Richter et al. reported that apoM gene expression could be regulated by HNF-1α. Mutant HNF-1α-/- mice completely lack expression of apoM in liver and kidney. Serum apoM levels in HNF-1α+/- mice are reduced by 50% compared with wild-type animals. By analyzing the apoM promoter and identifying a conserved HNF-1 binding site, they showed that HNF-1α is a potent activator of apoM promoter, that a specific mutation in the HNF-1 binding site abolished transcriptional activation of apoM gene. HNF-1α protein can bind to the HNF-1 binding site of apoM promoter *in vitro *[20]. Liang and Tall reported that leptin up-regulated mRNA level of apoM in *ob/ob *mice [21], suggesting that leptin could stimulate hepatic cells to produce apoM. Faber et al. reported that plasma concentration of apoM was similar in wild-type, LDL receptor-deficient and apoE deficient mice but was reduced by 33% in apoA-I-deficient mice compared with the wide-type mice, which suggest a connection between apoM and apoA-I metabolism [3]. Xu et al., found that in both liver and kidney, expression of apoM was significantly lower in leptin deficient *ob/ob *mice and in leptin-receptor deficient *db/db *mice than in control mice. Furthermore, leptin administration significantly increased plasma apoM levels and apoM mRNA levels in liver and in kidney in *ob/ob *mice [22]. It is concluded that both leptin and leptin-receptor are essential for the apoM expression, indicating that leptin is a physiological regulating factor on apoM synthesis *in vivo*.

## Physiopathology and potential clinical importance of apoM

Xu et al. investigated the relationship between plasma apoM levels and leptin levels, body mass index (BMI), fasting glucose, fasting insulin as well as lipoprotein concentrations in females displaying a wide range in BMI (18.9–57.1 kg/m^2^, n = 51). In univariate analysis, apoM correlated significantly to leptin (r = 0.54, P < 0.001), BMI (r = 0.70, P < 0.001), fasting insulin (r = 0.33, P = 0.025), total cholesterol (r = -0.41, P = 0.016), and LDL-cholesterol (r = -0.39, P = 0.018). The correlations between apoM and cholesterol and between apoM and leptin remained significant after adjustment for the influence of BMI. Forward stepwise multiple regressions when leptin, BMI, insulin and cholesterol were entered in a model as independent variables and apoM as the dependent variable showed that cholesterol and leptin were independent predictors of circulating apoM. These two parameters yielded an r^2 ^of 0.28, thereby explaining approximately 30% of the variance in apoM. Hence, apoM is positively correlated to leptin and negatively correlated to cholesterol levels in humans [23]. Richter et al. measured apoM levels in the serum of nine HNF-1α /maturity-onset diabetes of the young (MODY3) patients, nine normal matched control subjects (HNF-1α +/+), and nine HNF-4α /MODY1 subjects. Serum levels of apoM were significantly decreased in HNF-1α /MODY3 subjects when compared with control subjects as well as with HNF-4α /MODY1 subjects, indicating that HNF-1α haploinsufficiency rather than hyperglycemia is the primary cause of decreased serum apoM protein concentrations. Thus, serum levels of apoM may be a useful serum marker for the identification of MODY3 patients [20]. Alzheimer's disease (AD) is a complex, multifactor disorder, probably resulting from an interaction between environmental and genetic factors [24-26]. Increasing evidence points to a link between cholesterol turnover and AD [27,28], suggesting that genes implicated in brain cholesterol homeostasis may be potential candidate genes for AD. It is well known that apoE genotype and apoE receptor are related to AD [28,29]. With this background, Kabbara et al examined association of apoM with the risk of developing AD. It is excluded apoM as a genetic determinant of AD in a large French case control population [30].

## Conclusion

In conclusion, apoM is a novel HDL apolipoprotein. Like apoB apoM could be regulated by several cytokines *in vivo *and *in vitro*. HNF-1α is one of the most important activator of apoM gene promoter. Plasma apoM concentration is positively correlated to leptin levels and negatively related to plasma cholesterol levels. Both leptin and leptin receptor are essential for apoM expression *in vivo*. Plasma apoM levels may be used as the marker for identification of MODY3. The detailed relationship between apoM and MODY3 as well as obese needs further investigation.
